# LncRNA FAM83H-AS1 promotes triple-negative breast cancer progression by regulating the miR-136-5p/metadherin axis

**DOI:** 10.18632/aging.102832

**Published:** 2020-02-17

**Authors:** Chunyong Han, Yiwei Fu, Ni Zeng, Jian Yin, Qian Li

**Affiliations:** 1 Department of Breast Reconstruction, The Sino-Russian Joint Research Center for Oncoplastic Breast Surgery, Tianjin Medical University Cancer Institute and Hospital, National Clinical Research Center for Cancer, Key Laboratory of Cancer Prevention and Treatment of Tianjin, Tianjin Clinical Research Center for Cancer, Key Laboratory of Breast Cancer Prevention and Therapy, Tianjin Medical University, Ministry of Education, Tianjin 300060, China; 2 Tianjin Medical University Cancer Institute and Hospital, National Clinical Research Center for Cancer, Key Laboratory of Cancer Prevention and Treatment of Tianjin, Tianjin Clinical Research Center for Cancer, Key Laboratory of Breast Cancer Prevention and Therapy, Tianjin Medical University, Ministry of Education, Tianjin 300060, China; 3 Department of Cell Biology, Basic Medical College, Tianjin Medical University, Tianjin 300070, China; 4 Department of Respiratory, Tianjin Fifth Central Hospital, Tianjin 300457, China

**Keywords:** triple-negative breast cancer (TNBC), FAM83H-AS1, miR-136-5p, metadherin (MTDH)

## Abstract

In this study, we evaluated the function and regulation of the long non-coding RNA (lncRNA) FAM83H-AS1 in triple-negative breast cancer (TNBC). Our data show that the FAM83H-AS1 levels are increased in human TNBC cells and tissues. Proliferation, migration, and invasion of TNBC cells are decreased by FAM83H-AS1 suppression, but increased by FAM83H-AS1 overexpression. Bioinformatics analysis revealed that miR-136-5p is a potential target of FAM83H-AS1. MiR-136-5p expression is decreased in TNBC tissues, and its overexpression suppresses TNBC cell proliferation, migration, and invasion. MiR-136-5p suppression reverses the FAM83H-AS1 silencing-mediated inhibition of TNBC cell proliferation, migration, and invasion, suggesting that FAM83H-AS1 exerts its oncogenic effect by inhibiting miR-136-5p. Our data identify metadherin (MTDH) as the target gene of miR-136-5p, and demonstrate that the MTDH expression is increased in human TNBC tissues, which induces proliferation, migration, and invasion of TNBC cells. Importantly, our *in vivo* data show that FAM83H-AS1 also promotes tumor growth in TNBC mouse xenografts. Together, our results demonstrate that FAM83H-AS1 functions as an oncogenic lncRNA that regulates miR-136-5p and MTDH expression during TNBC progression, and suggest that targeting the FAM83H-AS1/miR-136-5p/MTDH axis may serve as a novel therapeutic target in TNBC.

## INTRODUCTION

Triple-negative breast cancer (TNBC) cells lack expression of estrogen receptor, progesterone receptor, and human epidermal growth factor receptor 2 (HER2). TNBC accounts for approximately 15% of invasive breast cancers, and is the most aggressive breast cancer subtype with a poor prognosis. While many patients with other breast cancer subtypes have benefited from targeted therapies, due to the lack of a definitive molecular therapeutic target, there have been no specific targeted drugs available for patients with TNBC. Conventional chemotherapy remains the standard of care for patients with advanced TNBC [1, 2]. Thus, it is imperative to identify the underlying mechanisms, and develop diagnostic and prognostic biomarkers for TNBC treatment.

Long noncoding RNAs (lncRNAs) are transcripts longer than 200 nucleotides without a protein-coding potential [3]. LncRNAs exert pivotal roles in physiological and pathological processes, including organ development, cell fate, and stemness maintenance [4–7]. Dysregulation of lncRNAs has been implicated in cancer cell proliferation, invasion, and metastasis [8–10]. LncRNA GClnc1 promotes gastric cancer progression and is associated with a poor prognosis in gastric cancer [10], while Lnc-UCID promotes hepatocellular carcinoma (HCC) tumorigenesis and correlates with HCC progression [11]. Elevated lncRNA TTTY15 promotes prostate cancer progression through targeting miRNA let-7 [12], while lncRNA n384546 promotes thyroid papillary cancer cell proliferation, invasion, and migration by regulating miR-145-5p /AKT3 signaling [13].

Several dysregulated lncRNAs have been implicated also in breast cancer progression. For example, LINC00511 correlates with a poor prognosis in breast cancer, and promotes breast cancer stemness and tumorigenesis via miR-185-3p/E2F1 [14]. LncRNA DANCR promotes breast cancer progression by regulating miR-216a-5p [15]. FOXD2-AS1 facilitates breast cancer cell proliferation, migration, and invasion by regulating miR-150-5p/PFN2 [16]. Moreover, the lncRNAs HCP5 [17], HOTAIR [18], NRAD1 [19], MIR503HG [20], and NAMPT-AS [21] are dysregulated and involved in TNBC progression. However, the function of the lncRNA FAM83H antisense RNA 1, FAM83H-AS1, is still unknown in TNBC.

FAM83H-AS1, also known as onco-lncRNA-3, is located on chromosome 8 (8q24.3), and has 2743 base pairs. FAM83H-AS1 has been shown to function as an oncogene in several human cancers. For example, FAM83H-AS1 is associated with worse survival rates in cervical cancer patients, and its inhibition decreases proliferation, migration, and survival of cervical cancer cells [22]. FAM83H-AS1 promotes radio-resistance and metastasis via targeting HuR protein in ovarian cancer [23]. FAM83H-AS1 also contributes to bladder cancer cell proliferation, migration, and invasion [24], and to glioma progression by epigenetically silencing CDKN1A (p21) [25]. FAM83H-AS1 correlates with poor prognosis in colorectal cancer, and promotes colorectal cancer cell proliferation by targeting the Notch signaling [26]. Moreover, FAM83H-AS1 contributes to lung cancer progression via regulating the MET/EGFR signaling [27]. In breast cancer, Yang et al have found that FAM83H-AS1 is upregulated in the luminal subtype of breast cancer [28]. In addition, the expression of FAM83H-AS1 is increased and correlates with poor survival rates in patients with early-stage breast cancer [29]. However, the function and the underlying mechanisms of FAM83H-AS1 in TNBC progression remain unknown.

In the present study, we investigated the role and the regulation of FAM83H-AS1 during TNBC progression. Our results demonstrate that the expression of FAM83H-AS1 is increased in TNBC cells and tissues, and promotes TNBC cell proliferation, migration, and invasion by regulating miR-136-5p and metadherin (MTDH) expression. These data indicate that the FAM83H-AS1/miR-136-5p/MTDH signaling might represent a potential therapeutic target in managing TNBC.

## RESULTS

### FAM83H-AS1 is upregulated in human TNBC tissues

First, we analyzed the expression of FAM83H-AS1 in human breast cancer tissues. Online data from the Gene Expression Profiling Interactive Analysis 2 (GEPIA2) (http://gepia2.cancer-pku.cn/#index) database showed that the expression of FAM83H-AS1 is increased in different types of cancer, including breast cancer (Supplementary Figure 1A, 1B, *p* < 0.05). Moreover, high FAM83H-AS1 levels are associated with a poor overall survival of breast cancer patients (Supplementary Figure 1C, 1D). The data analysis from cBioPortal revealed that 21% of breast cancer samples contain gene amplification of FAM83H-AS1 (Supplementary Figure 1E).

**Figure 1:**
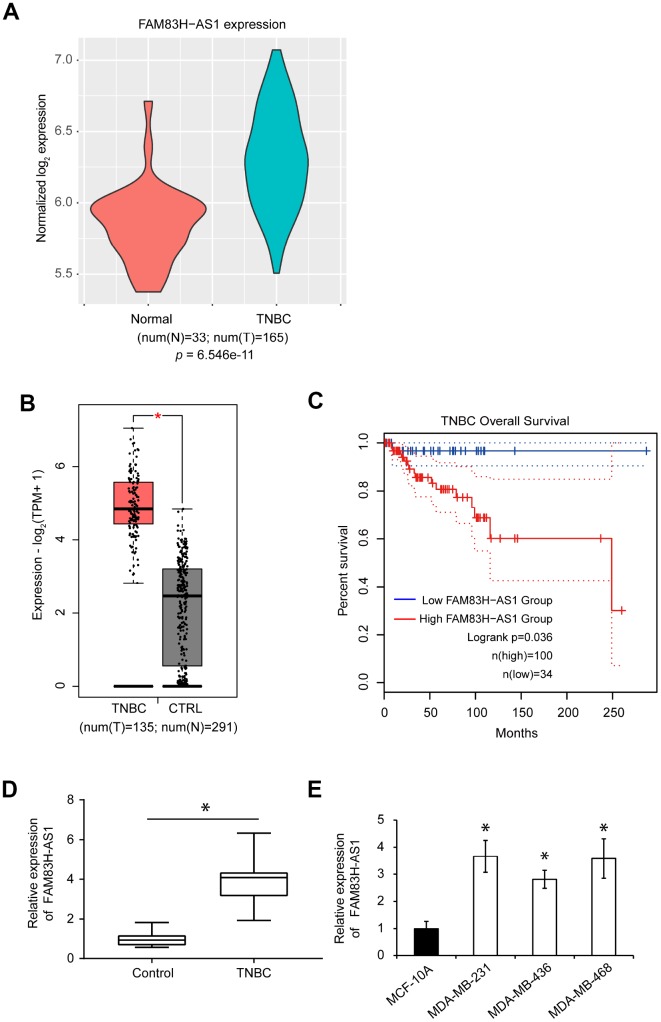
**FAM83H-AS1 is upregulated in TNBC tissues and predicts worse overall survival**. (**A**) Expression profiles of FAM83H-AS1 in TNBC and normal breast tissues using the human lncRNA microarray dataset GSE76250. The *p* value was calculated by Wilcoxon rank-sum test. (**B**) Expression profiles of FAM83H-AS1 in TNBC and normal breast tissues using the GEPIA 2 dataset. (**C**) Overall survival rates in low and high FAM83H-AS1 expression groups in TNBC patients using the GEPIA2 dataset. (**D**) qRT-PCR of FAM83H-AS1 expression in human TNBC and adjacent control tissues. (**E**) qRT-PCR of FAM83H-AS1 mRNA in MDA-MB-231, MDA-MB-436, MDA-MB-468, and MCF-10A cells.

Next, we analyzed the expression of FAM83H-AS1 in human TNBC tissues. The human lncRNA microarray dataset GSE76250 (containing 165 TNBC samples and 33 paired normal breast tissues) was downloaded using the Affymetrix Human Transcriptome Array 2.0 platform to analyze the expression profile of FAM83H-AS1 between TNBC and normal breast tissues. The expression of FAM83H-AS1 was significantly upregulated in TNBC compared to normal tissues (Figure 1A). Analysis of the GEPIA2 database also showed that the expression of FAM83H-AS1 is increased in human TNBC compared to normal breast tissues (Figure 1B, *p* < 0.05). Moreover, the upregulated expression of FAM83H-AS1 was predictive of a poor overall survival in TNBC patients (Figure 1C, *p* < 0.05). Furthermore, qRT-PCR analysis confirmed the increased expression of FAM83H-AS1 in TNBC compared to adjacent normal tissues (Figure 1D, *p* < 0.05). In addition, analysis of FAM83H-AS1 expression in three different TNBC cell lines (MDA-MB-231, MDA-MB-436, and MDA-MB-468) showed that the FAM83H-AS1 levels are increased in TNBC cell lines compared to normal human mammary epithelial cell line MCF-10A (Figure 1E, *p* < 0.05).

### FAM83H-AS1 promotes proliferation, migration, and invasion in TNBC cells

**Figure 2:**
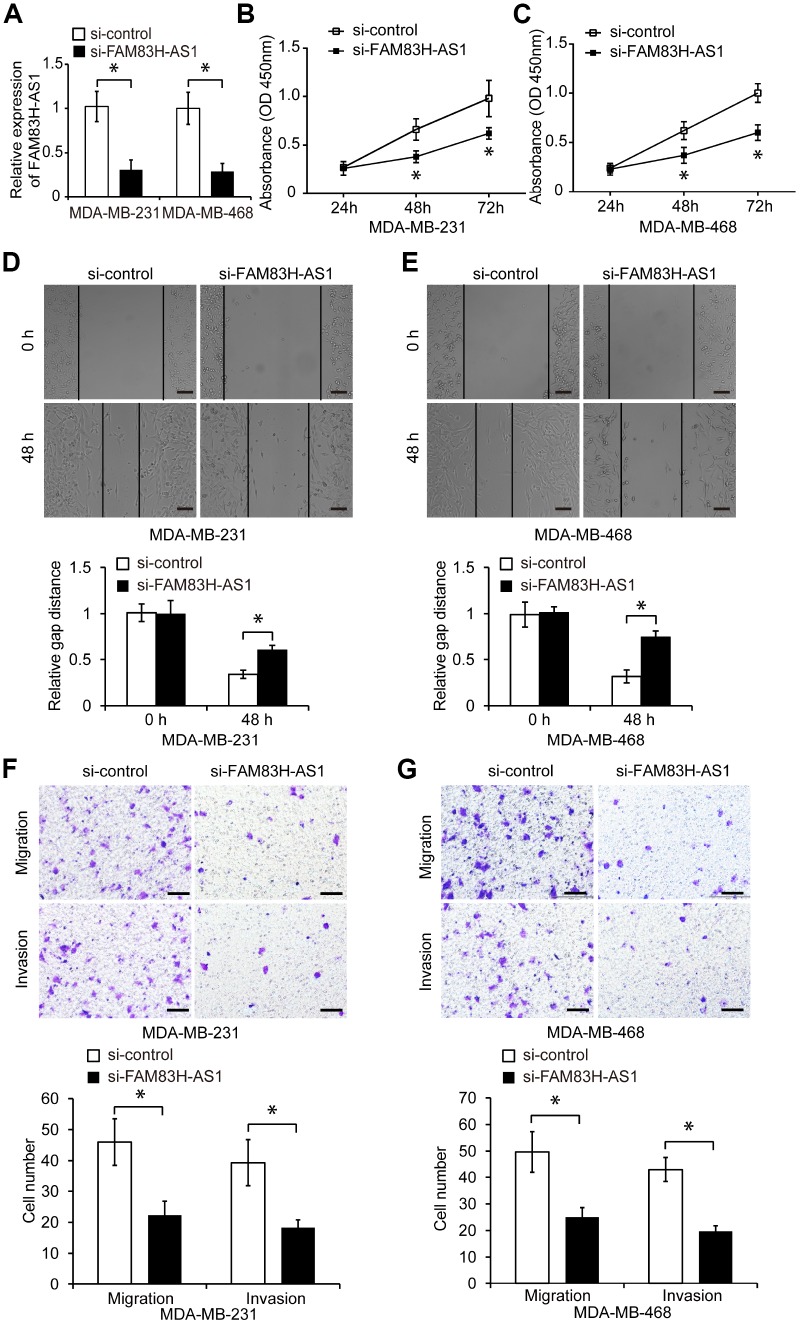
**FAM83H-AS1 suppression inhibits TNBC cell proliferation, migration, and invasion.** (**A**) qRT-PCR of FAM83H-AS1 expression in TNBC cells transfected with si-control or si-FAM83H-AS1 RNA. (**B**, **C**) Proliferation of TNBC cells transfected with si-control or si-FAM83H-AS1 RNA, analyzed by CCK8 assay. (**D**, **E**) Wound healing assay of the migration capacity of MDA-MB-231 and MDA-MB-468 cells transfected with si-control or si-FAM83H-AS1. (**F**, **G**) Migration and invasion of MDA-MB-231 and MDA-MB-468 cells transfected with si-control or si-FAM83H-AS1. Scale bars, 100 μm. * *p* < 0.05 compared to controls.

To investigate the function of FAM83H-AS1 in TNBC cells, we first suppressed the FAM83H-AS1 expression by specific siRNA in MDA-MB-231 and MDA-MB-468 cells (Figure 2A, *p* < 0.05). As shown in Figure 2B, 2C, FAM83H-AS1 suppression significantly reduced proliferation of TNBC cells measured by the CCK8 assay (*p* < 0.05). In addition, wound healing and transwell assays demonstrated that FAM83H-AS1 suppression markedly inhibited migration and invasion of TNBC cells compared to cells transfected with control siRNA (Figure 2D–2G, *p* < 0.05).

Next, we overexpressed FAM83H-AS1 in TNBC cells using the pcDNA-FAM83H-AS1 or empty vector pcDNA-control plasmids (Supplementary Figure 2A, *p* < 0.05). Overexpression of FAM83H-AS1 promoted proliferation of TNBC cells (Supplementary Figure 2B, 2C, *p* < 0.05), and increased their migration and invasion (Supplementary Figure 2D–2G, *p* < 0.05). These results indicate that FAM83H-AS1 promotes proliferation, migration, and invasion of TNBC cells *in vitro*.

### FAM83H-AS1 functions as a sponge for miR-136-5p in TNBC cells

**Figure 3:**
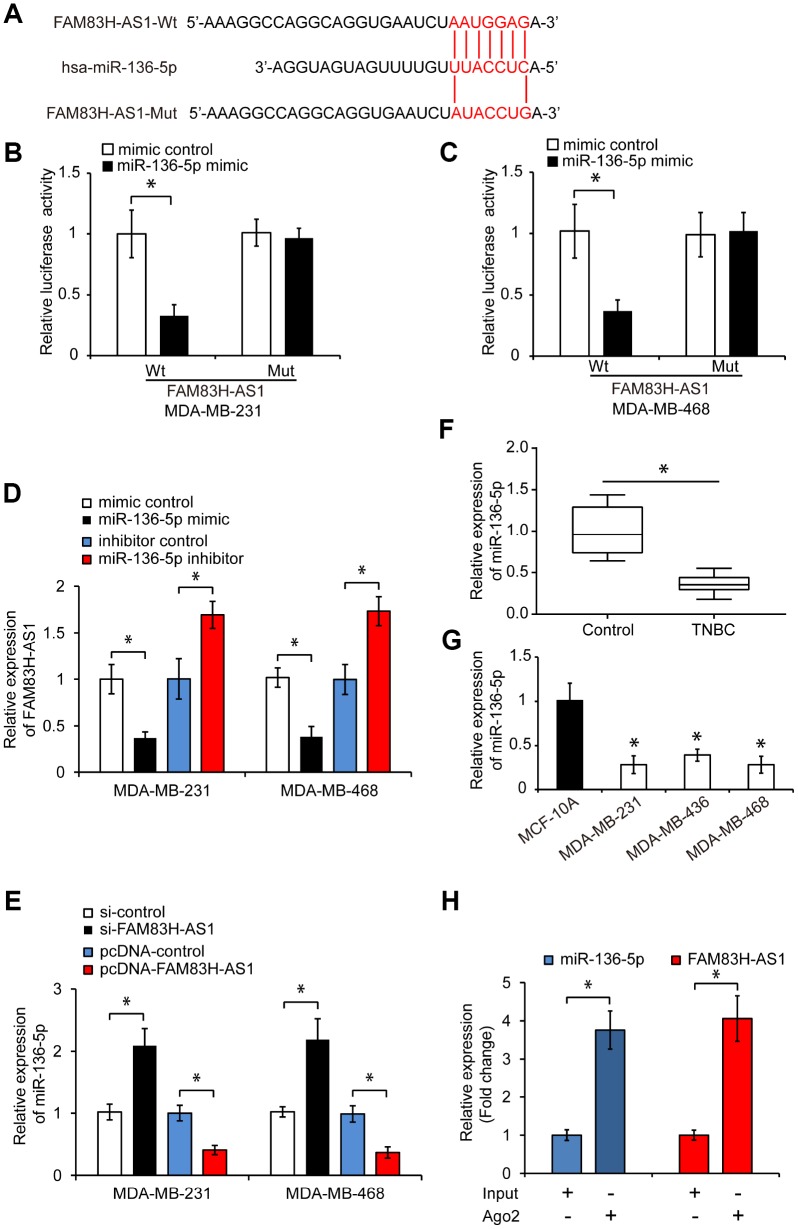
**FAM83H-AS1 functions as a sponge for miR-136-5p.** (**A**) Predicted binding site of miR-136-5p in the FAM83H-AS1 sequence and mutated nucleotides. (**B**, **C**) Overexpression of miR-136-5p repressed the luciferase activity in TNBC cells transfected with FAM83H-AS1-Wt assessed by luciferase reporter assays. (**D**) Relative FAM83H-AS1 expression in TNBC cells transfected with mimic control, miR-136-5p mimic, inhibitor control, or miR-136-5p inhibitor. (**E**) Relative miR-136-5p expression in TNBC cells transfected with si-control, si-FAM83H-AS1, pcDNA-control, or pcDNA-FAM83H-AS1. (**F**) qRT-PCR analysis of miR-136-5p expression in human TNBC and adjacent control tissues. (**G**) qRT-PCR analysis of miR-136-5p levels in TNBC cell lines MDA-MB-231, MDA-MB-436, and MDA-MB-468, and a control breast epithelial cell line MCF-10A. (**H**) RIP assay demonstrating the enrichment of FAM83H-AS1 and miR-136-5p. * *p* < 0.05 compared to controls.

To explore the mechanisms underlying the pro-oncogenic effects of FAM83H-AS1 in TNBC cells, we searched for potential FAM83H-AS1-targeted miRNAs using the LncBase Predicted v.2 bioinformatics database. We found that miR-136-5p was a potential target of FAM83H-AS1 (Figure 3A). To confirm this prediction, we constructed a luciferase reporter containing wild-type (Wt) or mutated (Mut) binding sites for miR-136-5p in FAM83H-AS1. As shown in Figure 3B and 3C, miR-136-5p overexpression significantly inhibited the luciferase activity in TNBC cells transfected with FAM83H-AS1-Wt, but not in FAM83H-AS1-Mut transfected cells (*p* < 0.05). In addition, overexpression of miR-136-5p significantly inhibited the FAM83H-AS1 expression, while miR-136-5p knockdown promoted the FAM83H-AS1 expression in TNBC cells (Figure 3D, *p* < 0.05). Moreover, FAM83H-AS1 knockdown increased the miR-136-5p expression, while FAM83H-AS1 overexpression decreased the miR-136-5p expression in TNBC cells (Figure 3E, *p* < 0.05). Analysis of miR-136-5p levels in human TNBC cells and tissues by qRT-PCR revealed that the miR-136-5p expression is reduced in TNBC tissues and cell lines (Figure 3F–3G, *p* < 0.05). In addition, RNA immunoprecipitation demonstrated that FAM83H-AS1 and miR-136-5p were highly enriched in Ago2-containing beads compared to controls (Figure 3H, *p* < 0.05), indicating that FAM83H-AS1 directly binds to miR-136-5p. Together, these data suggest that FAM83H-AS1 functions as a sponge for miR-136-5p in TNBC cells.

### MiR-136-5p inhibits proliferation, migration, and invasion in TNBC cells

**Figure 4:**
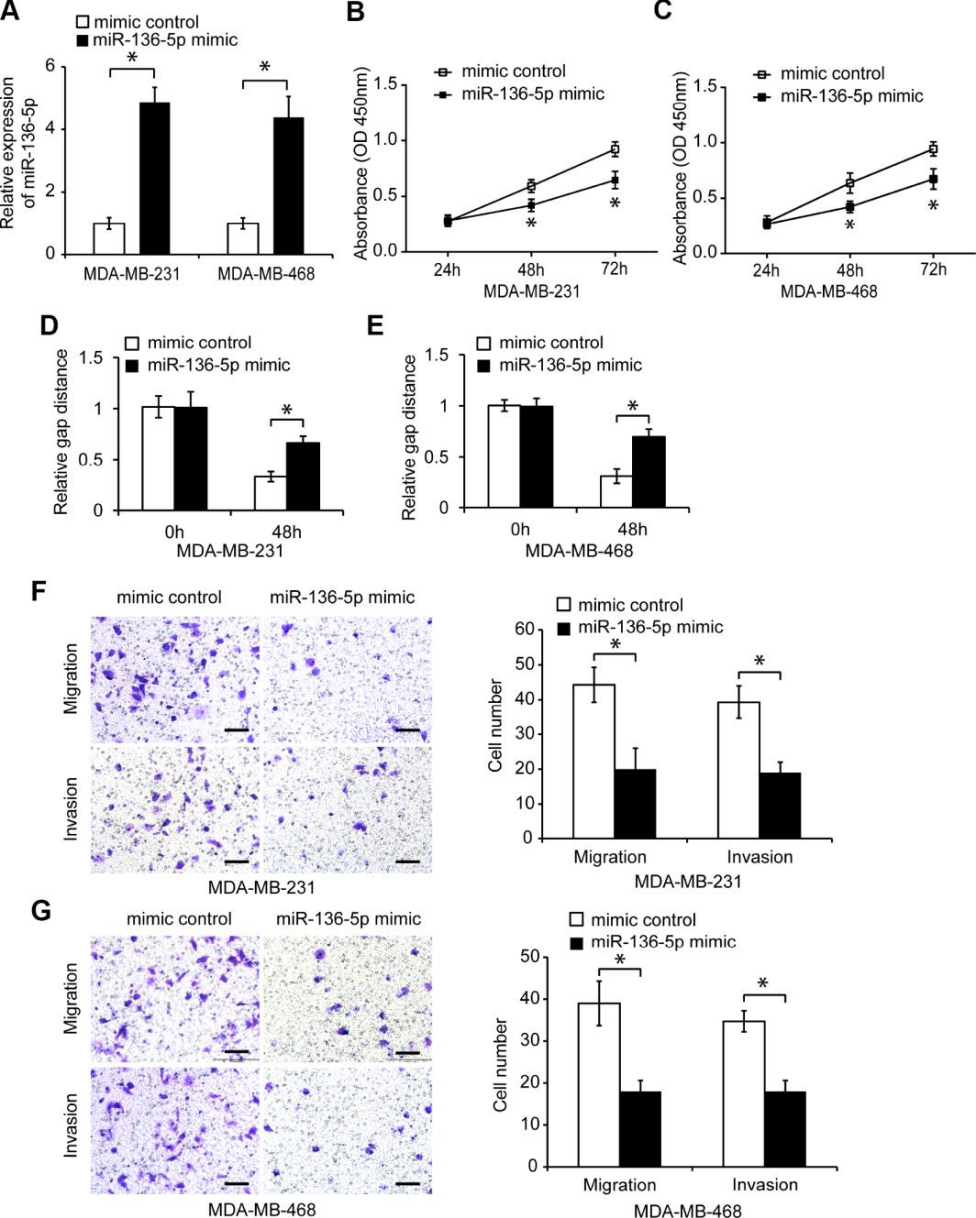
**Overexpression of miR-136-5p reduces TNBC cell proliferation, migration, and invasion.** (**A**) Relative miR-136-5p expression in TNBC cells transfected with mimic control or miR-136-5p mimic. (**B**, **C**) Proliferation of TNBC cells transfected with mimic control or miR-136-5p mimic, analyzed by CCK8 assay. (**D**, **E**) Wound healing assay of the migration capacity of TNBC cells transfected with mimic control or miR-136-5p mimic. (**F**, **G**) Migration and invasion of TNBC cells transfected with mimic control or miR-136-5p mimic, analyzed by transwell assays. Scale bars, 100 μm. * *p* < 0.05 compared to controls.

To investigate the function of miR-136-5p in TNBC cells, we first overexpressed miR-136-5p in MDA-MB-231 and MDA-MB-468 cells. MiR-136-5p mimic transfection significantly increased the endogenous miR-136-5p expression in TNBC cells (Figure 4A, *p* < 0.05), and reduced their proliferation (Figure 4B, 4C, *p* < 0.05). In addition, miR-136-5p overexpression significantly decreased migration and invasion of TNBC cells (Figure 4D–4G, *p* < 0.05). Conversely, transfection of miR-136-5p inhibitor decreased the expression of miR-136-5p in TNBC cells (Supplementary Figure 3A, *p* < 0.05), and promoted TNBC cell proliferation (Supplementary Figure 3B, 3C, *p* < 0.05). Moreover, the reduced miR-136-5p expression increased TNBC cell migration and invasion (Supplementary Figure 3D–3G, *p* < 0.05), indicating that miR-136-5p acts as a tumor suppressor in TNBC cells.

### FAM83H-AS1 exerts its pro-oncogenic function in TNBC cells by inhibiting miR-136-5p

**Figure 5:**
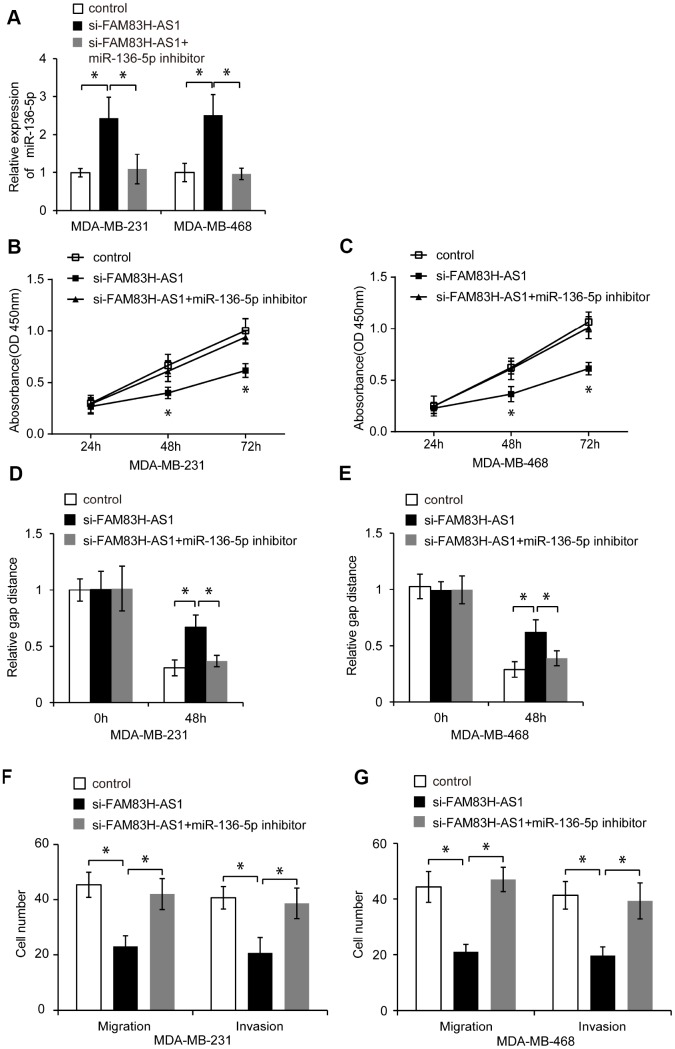
**FAM83H-AS1 promotes TNBC cell proliferation, migration, and invasion through inhibiting miR-136-5p.** (**A**) Relative miR-136-5p expression in TNBC cells transfected with control, si- FAM83H-AS1, and si-FAM83H-AS1+miR-136-5p inhibitor evaluated by qRT-PCR. (**B**, **C**) Proliferation of TNBC cells transfected with control, si-FAM83H-AS1, and si-FAM83H-AS1+miR-136-5p inhibitor, analyzed by CCK8 assay. (**D**, **E**) Wound healing assay of the migration of MDA-MB-231 and MDA-MB-468 cells transfected with control, si-FAM83H-AS1, and si-FAM83H-AS1+miR-136-5p inhibitor. (**F**, **G**) Migration and invasion of TNBC cells transfected with control, si-FAM83H-AS1, and si-FAM83H-AS1+miR-136-5p inhibitor, assessed by transwell assays. * *p* < 0.05 compared to controls.

To determine whether FAM83H-AS1 exerts its pro-oncogenic function in TNBC cells via inhibiting miR-136-5p, we first knocked down miR-136-5p expression in FAM83H-AS1-silenced TNBC cells (Figure 5A, *p* < 0.05), and then analyzed their proliferation, migration and invasion. As shown in Figure 5B–5G, miR-136-5p suppression blocked the suppressive effect of FAM83H-AS1 knockdown on TNBC cell proliferation, migration, and invasion (*p* < 0.05). These results show that FAM83H-AS1 promotes cell proliferation, migration, and invasion through inhibiting miR-136-5p in TNBC cells.

### MiR-136-5p suppresses metadherin expression in TNBC cells

**Figure 6:**
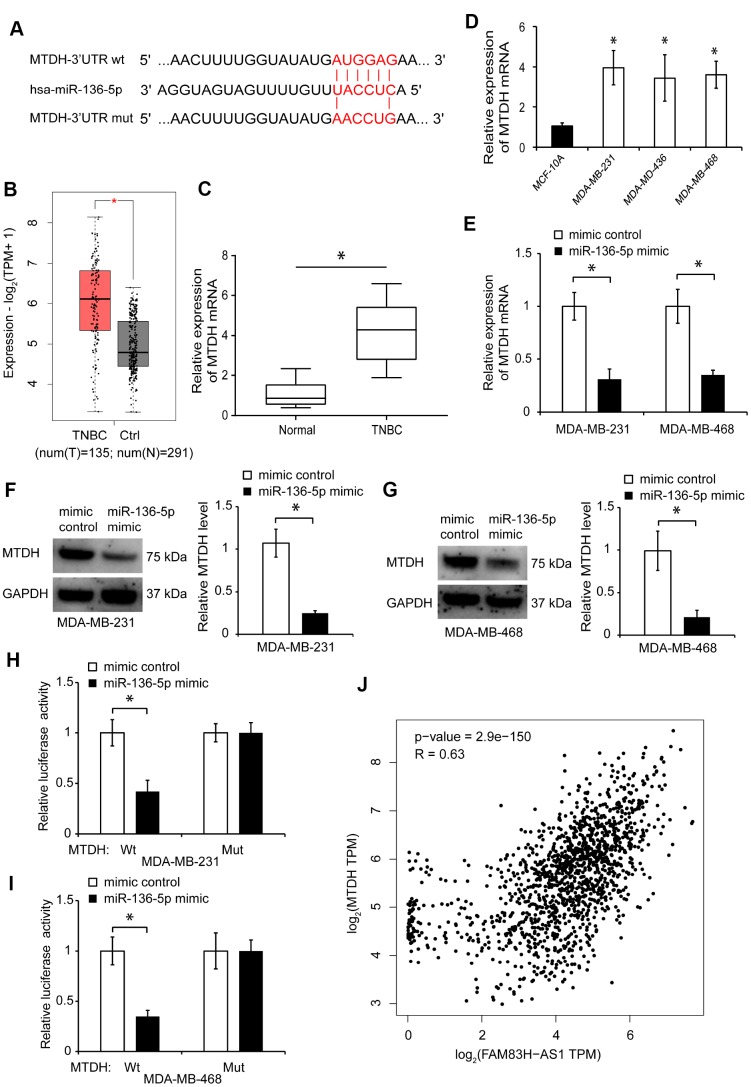
**MiR-136-5p suppresses MTDH expression in TNBC cells.** (**A**) Predicted binding site of miR-136-5p in MTDH sequence and mutated nucleotides. (**B**) MTDH expression in TNBC tissues analyzed using GEPIA 2 dataset. (**C**) qRT-PCR analysis of MTDH expression in human TNBC tissues. (**D**) qRT-PCR analysis of MTDH expression in MDA-MB-231, MDA-MB-436, MDA-MB-468, and MCF-10A cells. (**E**) MTDH expression in TNBC cells transfected with mimic control or miR-136-5p mimic. (**F**, **G**) Western blot analysis of MTDH protein levels in TNBC cells transfected with mimic control or miR-136-5p mimic. (**H**, **I**) Overexpression of miR-136-5p represses the luciferase activity in TNBC cells transfected with MTDH-Wt, evaluated by luciferase reporter assays. (**J**) Correlation between FAM83H-AS1 and MTDH expression in breast cancer tissues analyzed by Spearman’s rank test using the GEPIA 2 dataset. * *p* < 0.05 compared to controls.

We searched for the potential target genes of miR-136-5p through bioinformatics analysis using miRPathDB and miRTarBase. We found that two genes, BCL2 and metadherin (MTDH), were the potential targets of miR-136-5p (Supplementary Figure 4A). However, since transfection with miR-136-5p mimic did not have any significant effect on BCL2 expression in TNBC cells (Supplementary Figure 4B), the BCL2 gene was excluded from further analysis. Using bioinformatics analysis, we found that there is a putative binding site for miR-136-5p in the 3’-UTR of MTDH mRNA (Figure 6A). Online data analysis using cBioPortal and GEPIA 2 showed an increased MTDH expression in breast cancer tissues, including the TNBC subtype (Supplementary Figure 5A–5C, Figure 6B, *p* < 0.05). The increased expression of MTDH in breast cancer tissues was validated by gene mutation analysis using the cBioPortal data (Supplementary Figure 5D, 5E). The elevated MTDH expression was associated with poor overall survival rates in breast cancer patients, based on the cBioPortal and GEPIA 2 data analyses (Supplementary Figure 5F, 5G).

In addition, using qRT-PCR, we found that the MTDH gene expression is increased in TNBC tissues and cell lines (Figure 6C, 6D, *p* < 0.05). Furthermore, we found that miR-136-5p overexpression inhibits the MTDH expression in TNBC cells (Figure 6E–6G, *p* < 0.05). To determine whether MTDH binds to miR-136-5p, we constructed luciferase reporters containing Wt or Mut miR-136-5p binding sites. Overexpression of miR-136-5p significantly inhibited the luciferase activity in TNBC cells transfected with MTDH-Wt, but not MTDH-Mut (Figure 6H, 6I, *p* < 0.05). Moreover, analysis of the GEPIA 2 database indicated that the expression of MTDH positively correlates with the level of FAM83H-AS1 in breast cancer tissues (Figure 6J). These findings indicate that miR-136-5p suppresses the MTDH expression in TNBC cells.

### MiR-136-5p exerts its tumor-suppressive function in TNBC cells by suppressing MTDH

We investigated whether miR-136-5p exerts its tumor-suppressive function in TNBC cells by suppressing the MTDH expression. To examine the function of MTDH in TNBC cells, we suppressed the endogenous MTDH expression by using MTDH specific si-RNA (Supplementary Figure 6A, *p* < 0.05). MTDH knockdown markedly suppressed proliferation (Supplementary Figure 6B, 6C, *p* < 0.05), migration, and invasion of TNBC cells compared to control siRNA (Supplementary Figure 6D–6G, *p* < 0.05).

**Figure 7:**
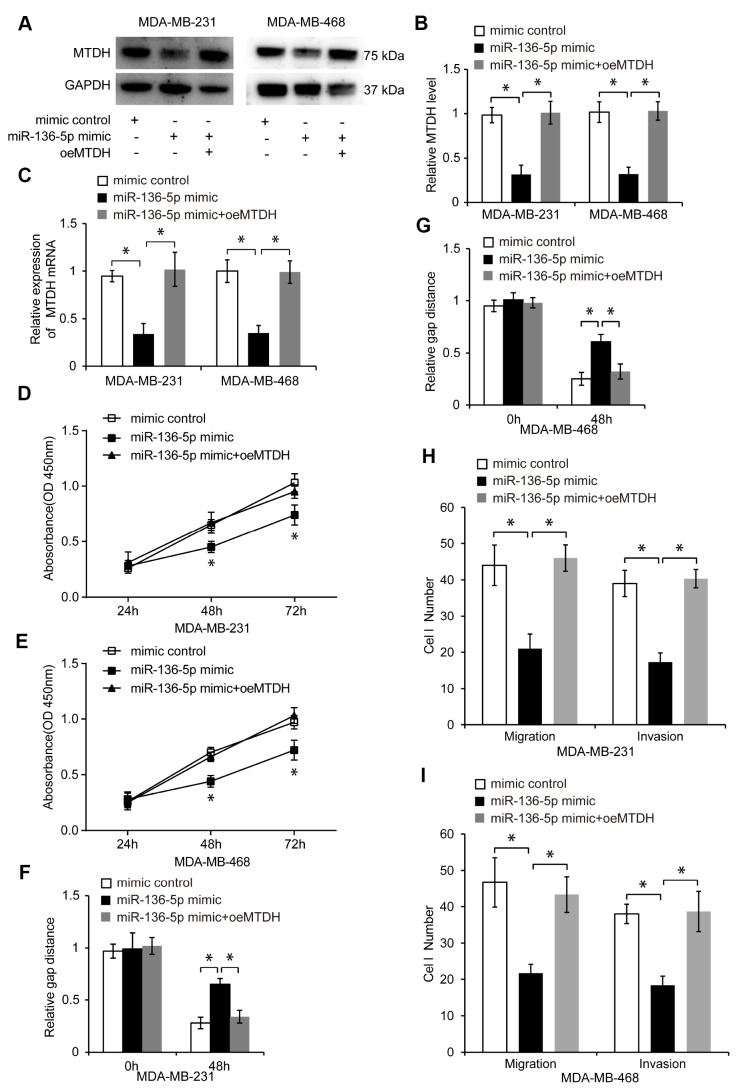
**MiR-136-5p inhibits proliferation, migration, and invasion of TNBC cells through suppressing MTDH**. (**A**, **B**) Western blot analyses in TNBC cells transfected with mimic control, miR-136-5p mimic, or miR-136-5p mimic plus oeMTDH. (**C**) Expression of MTDH in TNBC cells transfected with mimic control, miR-136-5p mimic, or miR-136-5p mimic plus oeMTDH. (**D**, **E**) CCK8 assay utilized to evaluate cell proliferation of TNBC cells transfected with mimic control, miR-136-5p mimic, or miR-136-5p mimic plus oeMTDH. (**F**, **G**) Wound healing assay used to determine migration of TNBC cells transfected with mimic control, miR-136-5p mimic, or miR-136-5p mimic plus oeMTDH. (**H**, **I**) Migration and invasion of TNBC cells transfected with mimic control, miR-136-5p mimic, or miR-136-5p mimic plus oeMTDH, analyzed by transwell assays. * *p* < 0.05 compared to controls.

Next, we overexpressed MTDH in miR-136-5p mimic-transfected TNBC cells via pcDNA-MTDH (oeMTDH) transfection. The transfection efficiency in control, miR-136-5p mimic, and miR-136-5p mimic plus oeMTDH groups was analyzed by qRT-PCR and western blotting (Figure 7A–7C, *p* < 0.05). Elevated MTDH expression abolished the inhibitory effect of miR-136-5p overexpression on TNBC cell proliferation, migration, and invasion (Figure 7D–7I, *p* < 0.05), suggesting that miR-136-5p exerts its tumor-suppressive function in TNBC cells by suppressing the MTDH expression.

### FAM83H-AS1 promotes tumor growth in TNBC xenograft mouse model

**Figure 8:**
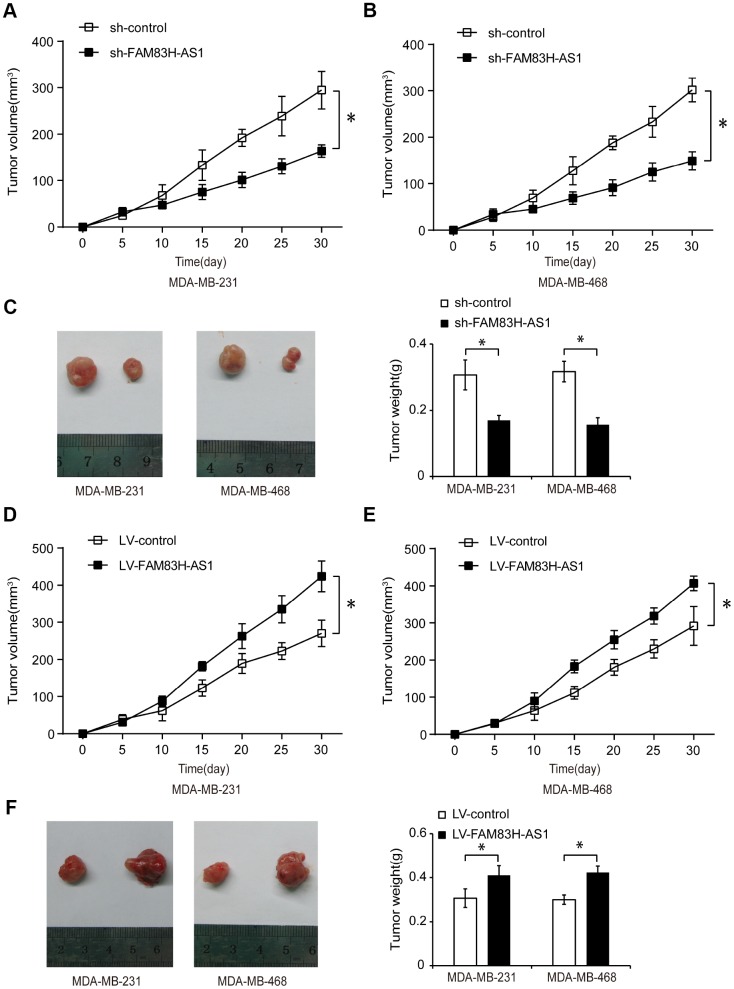
**FAM83H-AS1 promotes tumor growth in TNBC xenograft mouse model.** (**A**, **B**) Tumor volume measured every 5 days in mice injected with TNBC cells transfected with sh-control or sh-FAM83H-AS1. (**C**) Tumor weight in mice injected with TNBC cells transfected with sh-control or sh-FAM83H-AS1. (**D**, **E**) Tumor size measured in mice injected with TNBC cells transfected with LV-control or LV-FAM83H-AS1. (**F**) Tumor weight in mice injected with TNBC cells transfected with LV-control or LV-FAM83H-AS1. * *p* < 0.05 compared to controls.

To investigate the function of FAM83H-AS1 in regulating TNBC progression *in vivo,* we used a mouse TNBC xenograft model. TNBC cells were transfected with sh-control, sh-FAM83H-AS1, LV-control, or LV-FAM83H-AS1, and subcutaneously injected into female nude mice. As shown in Figure 8A–8C, mice injected with sh-FAM83H-AS1 had reduced tumor volumes and weight compared to mice injected with sh-control (*p* < 0.05), suggesting that FAM83H-AS1 suppression inhibits the TNBC tumor growth *in vivo*. Moreover, the tumor volume and weight were higher in LV-FAM83H-AS1 transfection group than in LV-control group (Figure 8D–8F, *p* < 0.05), suggesting that FAM83H-AS1 overexpression promotes the TNBC tumor growth *in vivo*. These data indicate that FAM83H-AS1 promotes the TNBC progression *in vivo*.

## DISCUSSION

Dysregulation of lncRNAs plays a critical role in the development, progression, and prognosis in a variety of human cancers, including breast cancer [19, 21, 30]. However, the function and regulation of the lncRNA FAM83H-AS1 in TNBC remain largely unknown. In our present study, we have found that FAM83H-AS1 promotes TNBC cell proliferation, migration, and invasion *in vitro,* and induces TNBC tumor growth *in vivo*. MiR-136-5p is the downstream target of FAM83H-AS1. MiR-136-5p functions as a tumor suppressor in TNBC cells, and inhibits their proliferation, migration, and invasion. MiR-136-5p exerts its tumor suppressive effect by targeting the protein metadherin (MTDH). Our data show that FAM83H-AS1 promotes the TNBC progression via targeting the miR-136-5p/MTDH axis.

Previous studies have indicated that FAM83H-AS1 plays an important role in cancer progression. For instance, high expression of FAM83H-AS1 correlates with advanced tumor grade and FIGO stage, and predicts radio-resistance, metastasis risk, and poor overall survival in ovarian cancer patients [23, 31]. Upregulated FAM83H-AS1 expression is also associated with worse survival rates in human cervical cancer [22], bladder cancer [24] and lung cancer [27]. FAM83H-AS1 overexpression predicts short survival times, and promotes tumor cell proliferation via targeting the Notch signaling in colorectal cancer [26, 32]. Moreover, increased FAM83H-AS1 expression is associated with poor survival rates in patients with breast cancer [28, 29]. Our results demonstrate that FAM83H-AS1 promotes TNBC cell proliferation *in vitro,* and induces TNBC tumor growth in an *in vivo* xenograft model.

LncRNAs may exert their functions by targeting miRNAs as miRNA sponges [30, 33]. To explore the molecular mechanism by which FAM83H-AS1 promotes TNBC progression, we searched for the potential miRNA targets of FAM83H-AS1 in TNBC cells by using bioinformatics prediction. Interestingly, miR-136-5p was identified as a potential target for FAM83H-AS1; this was further confirmed by qRT-PCR, luciferase activity, and the RIP assays. MiR-136-5p was found to inhibit cell proliferation, migration and invasion in renal cell carcinoma [34], liver cancer - by regulating IRX5 [35], and in colon cancer, through targeting LRH-1/Wnt signaling [36]. MiR-136 also inhibits cell survival, proliferation, cancer stem cell spheroid formation, and tumor angiogenesis in paclitaxel-resistant ovarian cancer cells by targeting Notch3 [37]. A few studies have demonstrated that miR-136 plays an essential role in breast cancer progression. Yan et al have found that miR-136 inhibits migration and invasion of TNBC cells through targeting RASAL2 [38]. Additionally, miR-136 inhibits breast cancer progression via regulating the Wnt/β-catenin signaling [39]. Consistent with the above studies, we have found that miR-136-5p overexpression inhibits TNBC cell proliferation, migration, and invasion, while MiR-136-5p knockdown increases proliferation, migration, and invasion of TNBC cells. Our data indicate that FAM83H-AS1 promotes TNBC progression through inhibiting miR-136-5p.

In addition, our study shows that miR-136-5p exerts its suppressive function in TNBC by inhibiting the MTDH expression. MTDH, also known as AEG-1 (Astrocyte Elevated Gene 1) and Lyric, has been implicated in the development and progression of a variety of human cancers [40], including hepatic cancer [41], lung cancer [42], esophageal squamous cell carcinoma [43], glioblastoma multiforme [44], and gastric cancer [45]. Moreover, the MTDH gene is frequently amplified in breast cancer, and the increased MTDH expression is associated with increased aggressiveness [46, 47], paclitaxel resistance [48], and trastuzumab resistance [49] in breast cancer patients. Our study demonstrates that MTDH is the downstream target of miR-136-5p, and its expression is upregulated in human TNBC tissues and cells. Overexpression of MTDH is able to abrogate the inhibitory effect of miR-136-5p on TNBC cells. Thus, these data show that miR-136-5p inhibits TNBC cell proliferation, migration, and invasion through suppressing the MTDH expression.

In summary, our data demonstrate that FAM83H-AS1 functions as an oncogenic lncRNA during TNBC progression. FAM83H-AS1 promotes proliferation, migration, and invasion of TNBC cells by inhibiting the miR-136-5p levels, resulting in the increased MTDH expression. The FAM83H-AS1/miR-136-5p/MTDH axis may thus serve as a novel therapeutic target for TNBC patients.

## MATERIALS AND METHODS

### Patient tissues

Human primary TNBC tissues and the corresponding adjacent non-tumorous tissues were obtained from ten newly diagnosed TNBC patients (Stage I–IIA) between Jan 2018 and Jun 2018 at Tianjin Medical University Cancer Institute and Hospital (Tianjin, China). All specimens were snap-frozen and stored in liquid nitrogen for mRNA and protein extraction. None of these patients received any pre-operative chemotherapy, hormonotherapy, or radiotherapy. All patients signed the informed consent. The study protocol was approved by the Ethics Committee of Tianjin Medical University Cancer Institute and Hospital (P.R. China).

### Bioinformatics analysis

The online available transcriptome microarray gene expression data (GSE76250) were downloaded from the Gene Expression Omnibus database (GEO, https://www.ncbi.nlm.nih.gov/geo/query/acc.cgi?acc=GSE76250). The cohort (GSE76250) provided the microarray data of lncRNAs, contained 165 TNBC samples and 33 paired normal breast tissues. The microarray data were based on [HTA-2_0] Affymetrix Human Transcriptome Array 2.0. In addition, we analyzed online data using the Gene Expression Profiling Interactive Analysis 2 (GEPIA2) (http://gepia2.cancer-pku.cn/#index) [50] and cBioPortal (https://www.cbioportal.org/) databases.

### Cell culture and transfection

Human TNBC cell lines (MDA-MB-231, MDA-MB-436, and MDA-MB-468) and normal breast cell line (MCF-10A) were maintained in Dulbecco’s Modified Eagle’s Medium (DMEM; Gibco, Grand Island, NY, USA) supplemented with 10% fetal bovine serum (FBS; Gibco, Carlsbad, CA, USA), 100 U/ml penicillin and 100 U/ml streptomycin (Gibco). Cells were incubated at 37°C with 5% CO_2_ in a humidified atmosphere. siRNAs (si-control and si-FAM83H-AS1), miR-136-5p mimic, mimic control, miR-136-5p inhibitor and inhibitor control were obtained from Ribobio Co., Ltd. (Guangzhou, P.R. China). The pcDNA-FAM83H-AS1 and control pcDNA plasmids were constructed by GenePharma (Shanghai, China). Cell transfection was performed using Lipofectamine 2000 reagent (Invitrogen, Carlsbad, CA, USA) following the manufacturer’s instructions.

### CCK8 assay

TNBC cells were incubated for 24, 48, or 72 hours at 37°C, and cell proliferation was measured by the CCK-8 assay (CCK-8; Sigma-Aldrich, St. Louis, MO, USA) according to the manufacturer’s instructions.

### Transwell migration and invasion assays

Transfected TNBC cells were harvested for transwell invasion and migration assays. For migration assay, TNBC cells (1 × 10^5^ cells) were seeded into the upper side of chambers (Corning, Corning, NY, USA). For invasion assay, TNBC cells were seeded into the upper side of chambers pre-coated with Matrigel (BD Biosciences, San Jose, CA, USA). DMEM medium (500 μl) containing 10% FBS was added into the lower chambers. After incubation for 12 hours (migration assays) or 24 hours (invasion assays), cells in the upper chamber were wiped off, and cells in the lower chamber were fixed with 4% formaldehyde, and stained with crystal violet. After washing with PBS, the number of cells that migrated or invaded were counted and the photographs were taken under a light microscope.

### Wound healing assay

Wound healing assay was used to evaluate the cell migration. After transfection, cells were seeded into six well plates at 5×10^5^ cells/well until to about 90% confluence. Linear scratches were made on the cell layer using a 200 uL pipette tip. Then, cells were maintained in serum-free media for 48 hours. The wound healing process was observed under an inverted microscope (Olympus, Japan). The photographs of wounded areas were taken at 100×magnifcation.

### Quantitative RT-PCR (qRT-PCR)

Total RNA was extracted using TRIzol reagent (Invitrogen), and reverse transcribed into cDNAs by using a Reverse Transcription Kit with the M-MLV reverse transcriptase (Promega, Madison, WI, USA). Quantitative RT-PCR was utilized to determine the levels of FAM83H-AS1, miR-136-5p and MTDH with the SYBR Green detection system and the 7500 Real Time PCR System (Applied Biosystems). Glyceraldehyde-3-phosphate dehydrogenase (GAPDH) or U6 were used as internal controls for mRNA or miRNA, respectively. The relative expression was measured using 2^-ΔΔCt^ method. All experiments were conducted in triplicates.

### Luciferase reporter assay

Dual-Luciferase^®^ Reporter Assay System (Promega, Madison, WI, USA) was used to determine the luciferase activity 48 hours after transfection according to the manufacturer’s protocol. TNBC cells were co-transfected with a wild-type or mutant FAM83H-AS1 reporter plasmid, and either a miR-136-5p mimic or a miRNA mimic control using Lipofectamine 2000 (Invitrogen, Carlsbad, CA) in accordance with the manufacturer’s protocol.

### RNA immunoprecipitation (RIP)

Magna RNA immunoprecipitation (RIP) kit (Millipore, Billerica, USA) was used to conduct RIP assays in accordance with the manufacturer’s protocol. All cells were lysed using RIP lysis buffer and incubated with RIP immunoprecipitation buffer containing magnetic beads conjugated to human anti-Ago2 antibody (Abcam, Cambridge, MA) or control anti-IgG antibody. Co-precipitated RNAs were analyzed by qRT-PCR for the expression of FAM83H-AS1 and miR-136-5p.

### Western blot analysis

Total protein was extracted using RIPA lysis buffer (Beyotime Institute of Biotechnology, Beijing, China), and protein concentration was measured by BCA Protein Assay Kit (Beyotime Institute of Biotechnology, Beijing, China). Equal amounts of proteins (30 μg) were separated by 10% SDS-PAGE and transferred onto PVDF membranes (Millipore, Boston, MA, USA). The membranes were blocked with 5% (w/v) nonfat milk in Tris-buffered saline containing 0.1% Tween 20 (TBST) at room temperature, and incubated with primary antibody against MTDH (Cell Signaling Technology, Danvers, MA, USA) at 4°C overnight. Following washing with TBST, the membranes were incubated with horseradish peroxidase-conjugated secondary antibody (Santa Cruz Biotechnology, Dallas, TX, USA), and visualized using Enhanced Chemiluminescence Kit (GE Healthcare, Chicago, IL, USA).

### Lentivirus transduction and *in vivo* mouse model

The full-length sequence of FAM83H-AS1 was subcloned into pCDH-CMV-MCS-EF1-Puro lentiviral vector. The shRNA sequence specific to FAM83H-AS1 was subcloned into pLKO.1 lentiviral vector. The lentiviral vectors were co-transfected with the packaging vectors pAX8 and pCMV-VSVG into 293FT cells by Lipofectamine 2000. Two days later, viral supernatants were collected and used to infect cells. Forty-eight hours following infections, cells were selected with 1.5 μg/mL puromycin. TNBC cell lines were infected with either lentivirus-pLKO.1-sh-FAM83H-AS1 (sh-FAM83H-AS1), control lentivirus-shRNA (sh-control), lentivirus-FAM83H-AS1 (LV-FAM83H-AS1) or lentivirus-control virus (LV-control).

Nude mice (4~6 weeks old, female) were maintained under pathogen free conditions. All animal procedures were approved by the Animal Care Committee of Tianjin Medical University. For the tumor xenograft experiments, stable TNBC cells (2×10^6^) transfected with either sh-control, sh-FAM83H-AS1, LV-control or LV-FAM83H-AS1 were subcutaneously injected into mice (n = 3 per group). The tumor volume was measured every 5 days, and calculated by the formula: length × width^2^ × 0.5. At 30 days post-injection, the mice were euthanized and tumors were surgically isolated and photographed.

### Statistical analysis

Student’s unpaired t-test was used for comparisons between groups. All data are presented as mean ± standard deviation (SD). A value of *p* < 0.05 was regarded statistically significant. Statistical analysis was performed using the IBM SPSS 22.0 (IBM Corp., Armonk, NY, USA) and GraphPad Prism 8.0 (San Diego, CA, USA).

## Supplementary Material

Supplementary Figures
