# Supplementary material for: LncRNA FAM83H-AS1 promotes triple-negative breast cancer progression by regulating the miR-136-5p/metadherin axis
Source: Aging (Albany NY). 2020 Feb 17;12(4):3594–616. doi: 10.18632/aging.102832 (PMC7066879; doi:10.18632/aging.102832)
Supplement: Supplementary Figures [file aging-12-102832-s001..pdf]

SUPPLEMENTARY FIGURES

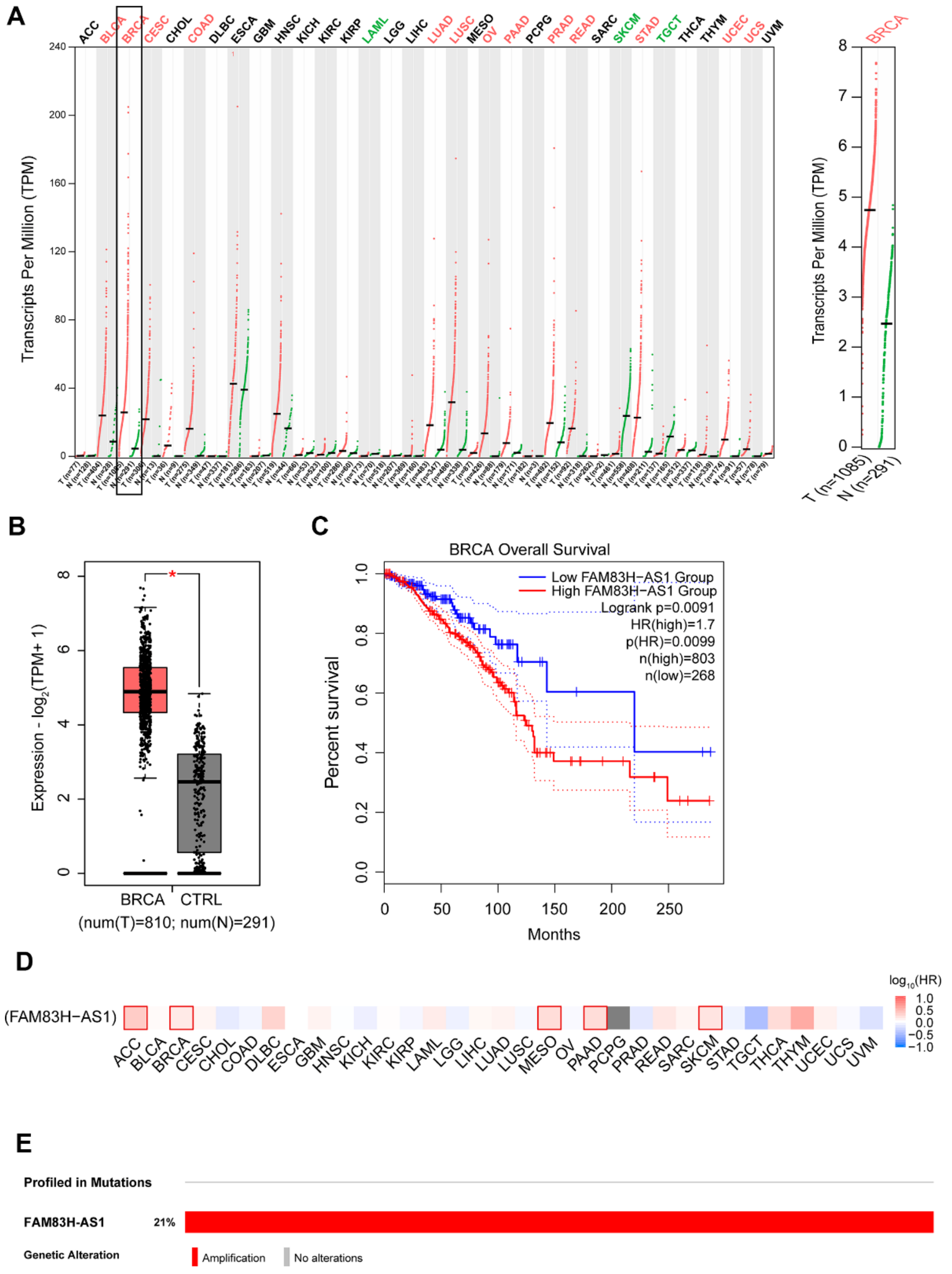

Supplementary Figure 1. FAM83H-AS1 is upregulated in breast cancer and predicts worse overall survival. (**A**) FAM83H-AS1 expression profile in a variety of cancers based on the GEPIA2 data. BRCA, breast cancer; T, tumor; N, normal. (**B**) FAM83H-AS1 expression profile between breast cancer and normal control based on GEPIA 2 data. BRCA, breast cancer; CTRL, control; (**C**) Overall survival analysis results between the low and the high FAM83H-AS1 expression groups in breast cancer patients based on GEPIA2 data. (**D**) Heat map which shows the effect of FAM83H-AS1 on patients' overall survival across multiple cancer types based on GEPIA2 data. (**E**) FAM83H-AS1 gene mutation profile in breast cancer based on the data from cBioPortal.

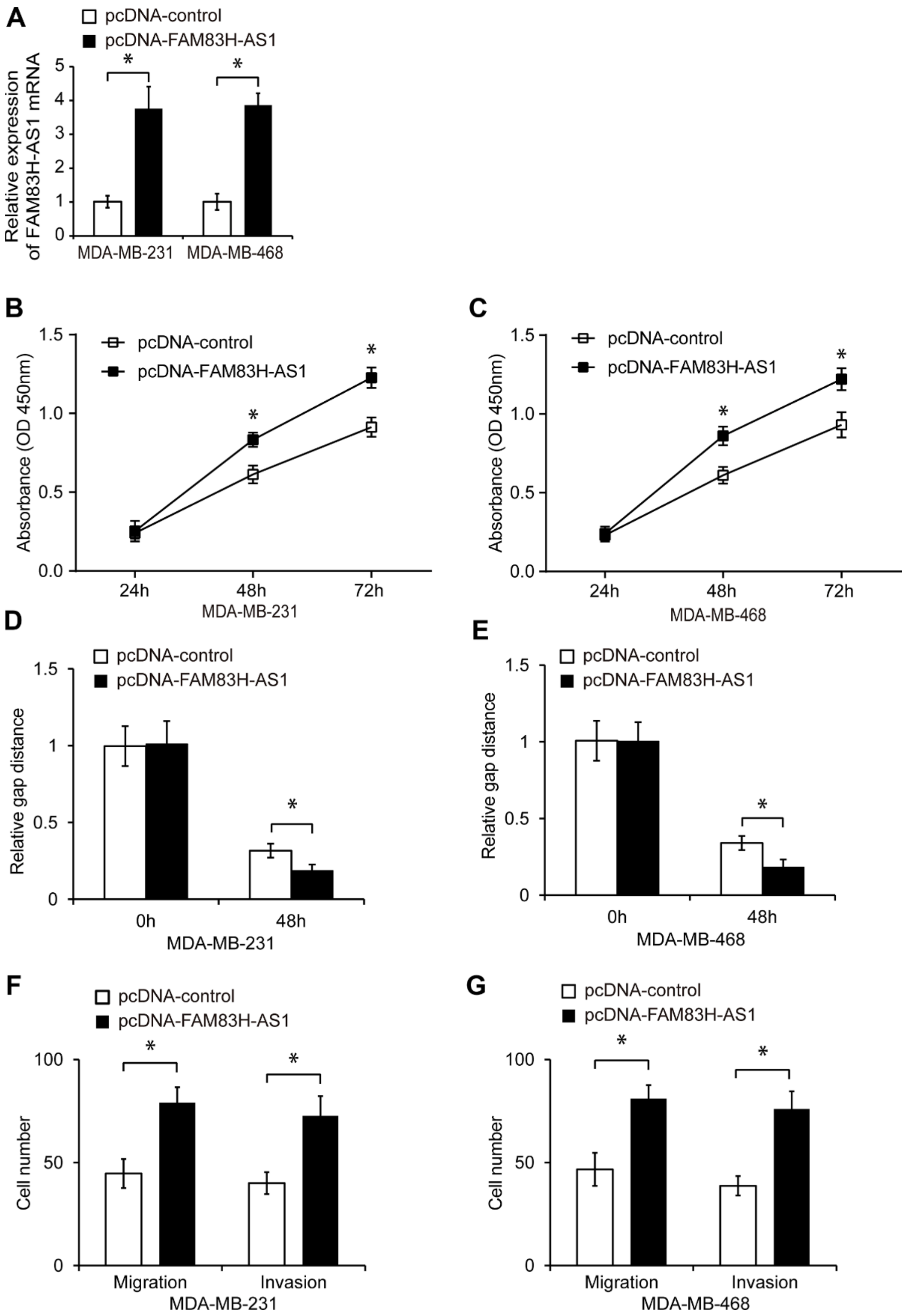

Supplementary Figure 2. Overexpression of FAM83H-AS1 markedly facilitated TNBC cell proliferation, migration and invasion. (**A**) Relative FAM83H-AS1 expression in TNBC cells transfected with pcDNA-control or pcDNA-FAM83H-AS1 evaluated by qRT-PCR. (**B**, **C**) CCK8 assays performed to determine the proliferation of TNBC cells transfected with pcDNA-control or pcDNA-FAM83H-AS1. (**D**, **E**) Wound healing assays used to determine the migration capacity of MDA-MB-231 and MDA-MB-468 cells transfected with pcDNA-control or pcDNA-FAM83H-AS1. (**F**, **G**) The migration and invasion capacities of MDA-MB-231 and MDA-MB-468 cells transfected with pcDNA-control or pcDNA-FAM83H-AS1 were assessed by transwell assays. * $p < 0.05$ compared to controls.

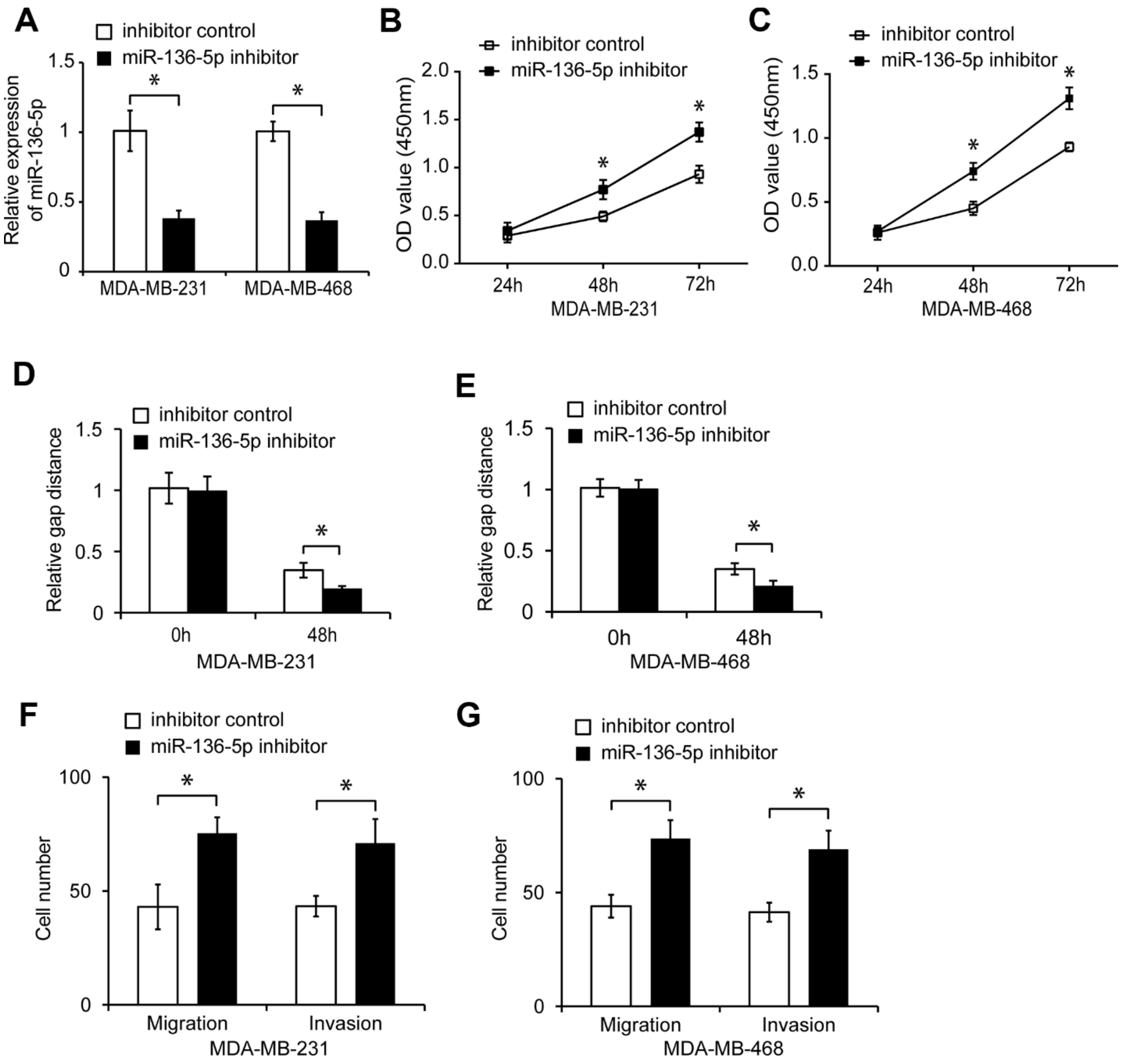

Supplementary Figure 3. MiR-136-5p knockdown promotes cell proliferation, migration and invasion in TNBC. (**A**) Relative miR-136-5p expression in TNBC cells transfected with inhibitor control or miR-136-5p inhibitor. (**B**, **C**) CCK8 assays used to determine the proliferation of TNBC cells transfected with inhibitor control or miR-136-5p inhibitor. (**D**, **E**) Wound healing assays used to determine the migration capacity of TNBC cells transfected with inhibitor control or miR-136-5p inhibitor. (**F**, **G**) The migration and invasion capacities of TNBC cells transfected with inhibitor control or miR-136-5p inhibitor were assessed by transwell assays. * $p < 0.05$ compared to controls.

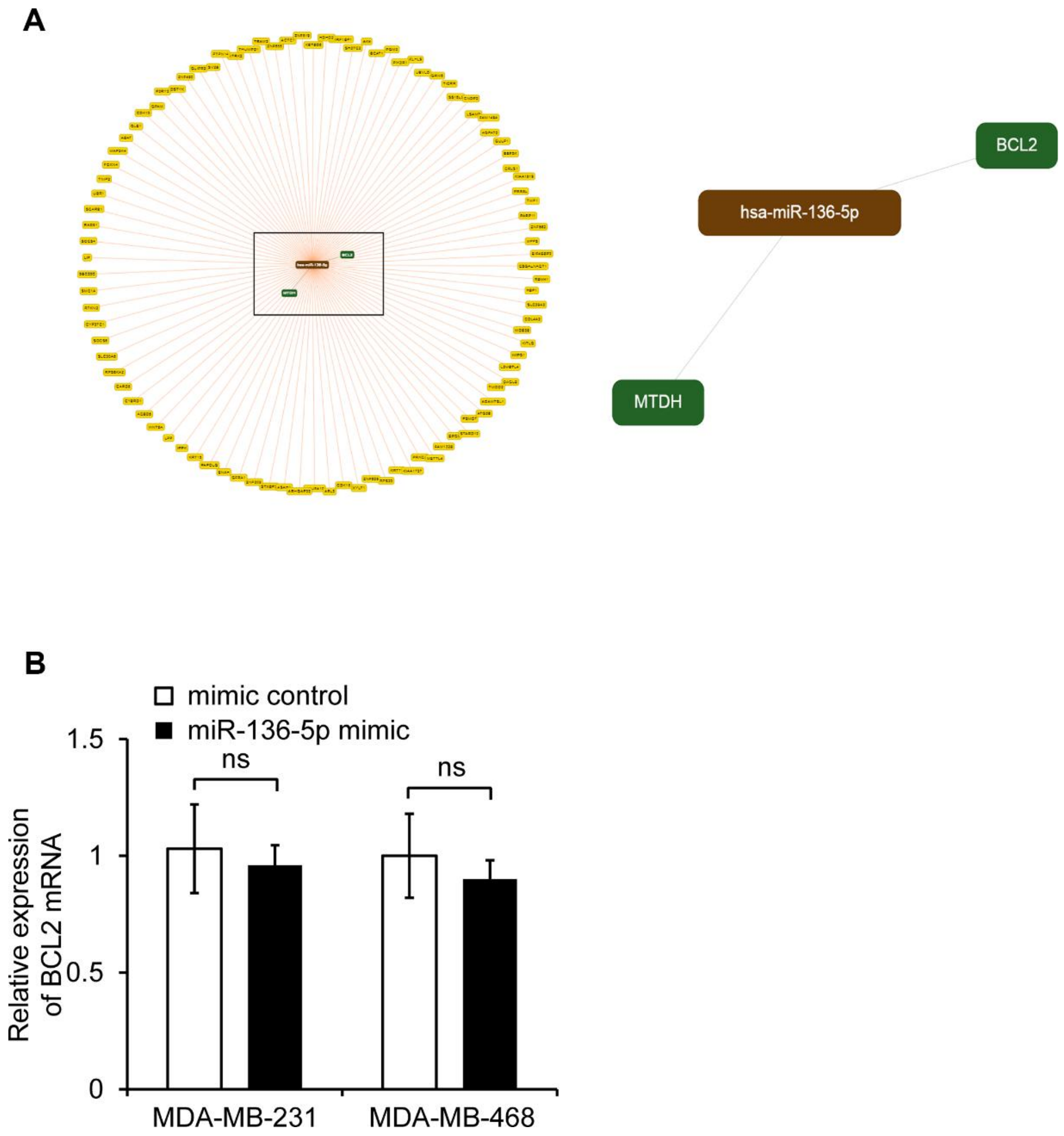

Supplementary Figure 4. BCL2 was not the target gene regulated by miR-136-5p in TNBC. (**A**) The potential target genes of miR-136-5p through bioinformatics analysis using miRPathDB and miRTarBase. (**B**) Relative BCL2 expression in TNBC cells transfected with either miR-136-5p mimic or mimic control. ns, not statistically significant.

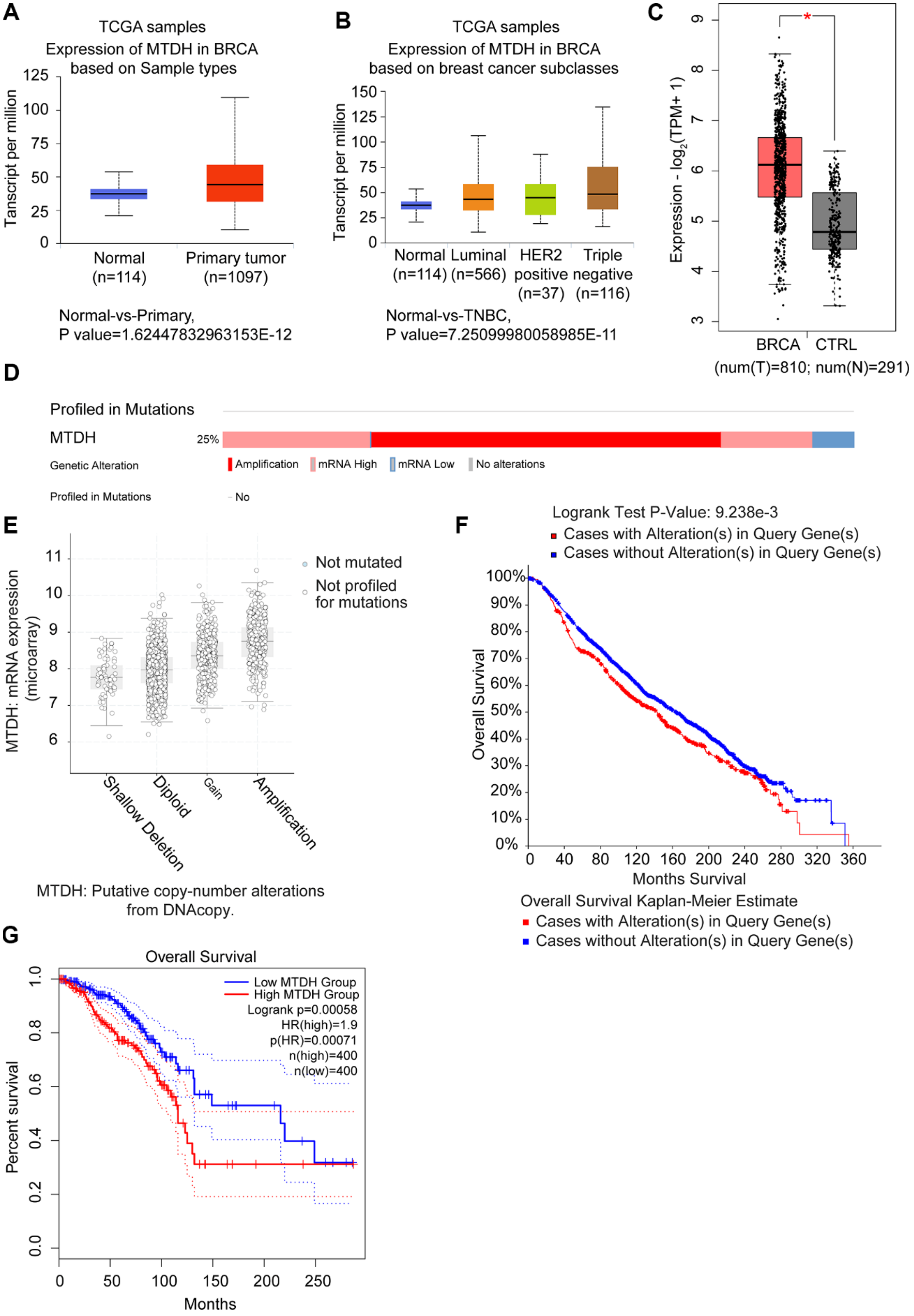

Supplementary Figure 5. MTDH is upregulated in breast cancer and predicts worse overall survival. (**A**, **B**) Online analysis of MTDH expression in breast cancer based on cBioPortal data. (**C**) Analysis of MTDH expression in breast cancer based on GEPIA 2 data. (**D**, **E**) MTDH gene mutation profile in breast cancer based on the data from cBioPortal. (**F**, **G**) Overall survival analysis results between the low and the high MTDH expression groups in breast cancer patients based on cBioPortal (**F**) and GEPIA2 (**G**) data.

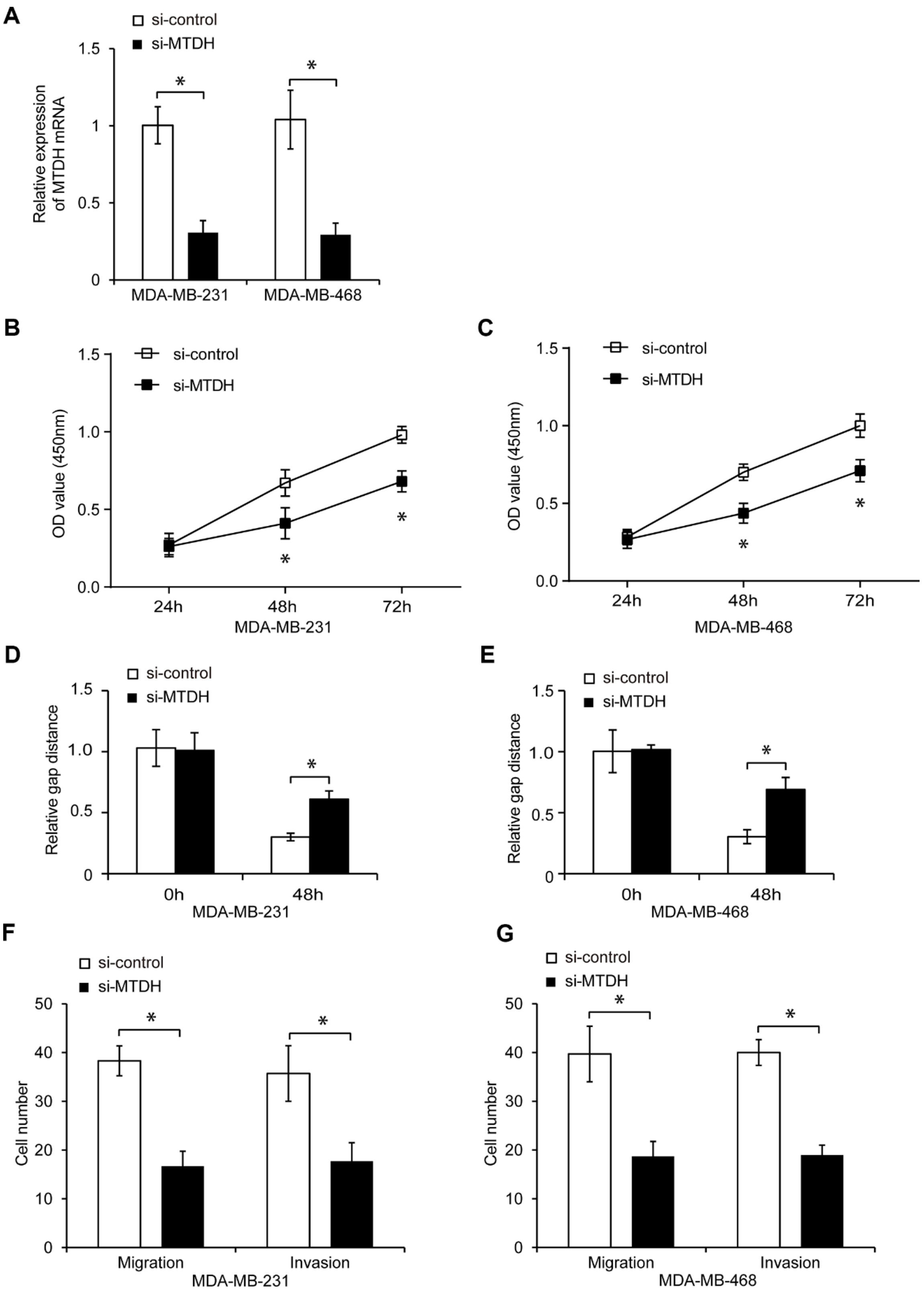

Supplementary Figure 6. MTDH knockdown inhibits cell proliferation, migration and invasion in TNBC. (**A**) Relative MTDH expression in TNBC cells transfected with si-control or si-MTDH. (**B**, **C**) CCK8 assays used to determine the proliferation of TNBC cells transfected with si-control or si-MTDH. (**D**, **E**) Wound healing assays used to determine the migration capacity of TNBC cells transfected with si-control or si-MTDH. (**F**, **G**) The migration and invasion capacities of TNBC cells transfected with si-control or si-MTDH were assessed by transwell assays. * $p < 0.05$ compared to controls.
